# Renal tubular epithelial cells injury induced by mannitol and its potential mechanism

**DOI:** 10.1080/0886022X.2017.1419973

**Published:** 2018-01-04

**Authors:** Jinwan Shi, Jiuzhan Qian, Hui Li, Hongjun Luo, Wenhong Luo, Zhexuan Lin

**Affiliations:** Bioanalytical Laboratory, Shantou University Medical College, Shantou, Guangdong, People’s Republic of China

**Keywords:** Cell apoptosis, cytoskeleton, cytotoxicity, HK-2 cell, mannitol, oxidative stress

## Abstract

Administration of mannitol with high dose could induce extensive isometric renal proximal tubular vacuolization and acute renal failure in clinic. We previously demonstrated that mannitol-induced human kidney tubular epithelial cell (HK-2) injury. The objective of our present work was to further study the cytotoxicity of mannitol in HK-2 cells and its potential mechanism. Cell viability was assessed by an MTT method. Cell morphological changes were observed. Furthermore, levels of malondialdehyde (MDA) and glutathione (GSH) were measured. Flow cytometry was performed to determine cell apoptosis by using Annexin V-FITC and PI. In addition, the F-actin of cells was labeled by FITC-Phalloidin for observation of cytoskeleton. The MTT assay displayed that the cell viability decreased significantly in a dose- and time-dependent manner. The morphological changes were observed, including cell membrane rapture and cell detachment. The GSH concentration in HK-2 cells decreased dramatically in mannitol treatment group, while MDA content increased significantly. The results of flow cytometry indicated that apoptotic percentages of HK-2 cells increased in 250 mmol/L mannitol treatment group. After treatment with 250 mmol/L mannitol for 48 h, HK-2 cells showed disorganization of cytoskeleton and even exhibited a totally destroyed cytoskeleton. Therefore, high dose of mannitol has a toxic effect on renal tubular epithelial cells, which might be attributed to oxidative stress, destroyed cellular cytoskeleton and subsequent cell apoptosis.

## 1. Introduction

Mannitol (C_6_H_14_O_6_), a six-carbon alcohol of the sugar mannose, is widely used as an osmotic diuretic agent to alleviate tissue edema [1] and to lower intraocular hypertension [2] and intracranial hypertension [3]. But in the past few years, the side effect of mannitol attracted a lot of attention, since maximum 200 g/d of mannitol would be administrated in clinic, which could cause nephrotoxicity or acute renal failure (ARF) in a proportion of patients [4,5]. Animal studies also revealed the occurrence of ARF after administration of large dose of mannitol [6,7]. Renal cortex congestion, swelling and vacuolization of tubular cells with substantial reduction in the proximal tubular lumen diameter were observed in rabbits after administration of large dose of mannitol [8,9]. Mannitol may induce extensive isometric renal proximal tubular vacuolization, intense afferent arteriolar constriction (particularly when combined with cyclosporine A) and ARF in higher doses [10–12]. Although in some animal researches, mannitol provides beneficial effects against contrast-induced nephropathy [13], the present meta-analysis and former studies based on human concluded the opposite [14,15]. In other words, prophylactic mannitol of high dose in some patients may be associated with significant toxicity [16]. Mannitol and similar solutes can enter tubular cells via pinocytosis, and to form vacuoles that subsequently fuse with each other and with lysosomes containing hydrolytic enzymes. It is at this level where lysosomal degradation and digestion can get impaired in diseases like diabetes mellitus and chronic kidney diseases that predisposes to mannitol-induced ARF. Although earlier and mild vacuolar changes are reversible, more overt damage can result in permanent injury to the renal tubules [17]. However, the mechanism of mannitol-induced ARF remains uncertain. In our previous experiment, we found that high doses of mannitol could inhibit renal tubular epithelial cells proliferation in a both time-dependent and dose-dependent manner [18]. Our present work was to further examine the cytotoxicity of mannitol on renal tubular epithelial cells *in vitro*, and investigate its potential mechanism.

## 2. Materials and methods

### 2.1. Reagents

Mannitol (CAS:69–65-8) and MTT (3-(4,5-dimethyl-2-thiazolyl)-2,5-diphenyl-2-H-tetrazolium bromide) kit were purchased from Aladdin (Shanghai, China), Dulbecco’s modified Eagle’s medium: nutrient mixture F-12 (DMEM-F12) was from Gibco (Invitrogen, Carlsbad, CA, USA), new-born calf serum (NCS) was from Tianhang (Hangzhou, China). The bicinchoninic acid (BCA) protein assay kit, 4',6-diamidino-2-phenylindole (DAPI) staining solution, malondialdehyde (MDA) assay kit were obtained from Beyotime Institute of Biotechnology, and glutathione (GSH) assay kit was purchased from Nanjing Jiancheng Bioengineering Institute, Nanjing, China. The Annexin V-FITC apoptosis detection kit was from Nanjing KeyGen Biotech Co. Ltd Nanjing, China. Trypsin-EDTA, Triton X-100, bovine serum albumin (BSA) were from Amresco (Solon, OH, USA). FITC-Phalloidin was obtained from Sigma (Chemical Co., St. Louis, MO, USA).

### 2.2. HK-2 cell culture

HK-2 cells were obtained from Chinese Academy of Medical Sciences. Cells were cultured in DMEM-F12 supplemented with 20% (v/v) NCS in a 100% humidified incubator with 5% CO_2_ at 37 °C. The cells were treated with 0.25% trypsin and 0.02% EDTA for passaging when they reached 80% confluence. The cells obtained on passage 3 and 4 were used for experiments.

### 2.3. Cell proliferation assay

Viability of HK-2 cells was determined by MTT assay. HK-2 cells were plated into 96-well plates at a density of 5 × 10^3^ cells/well in 0.1 mL complete medium. After overnight adhesion, the cells were treated with media containing mannitol (at concentrations of 0, 50, 100, 150, 200, 250, 300 and 400 mmol/L). In order to detect the time-dependent effect, the cells were treated for 4, 10, 24, 48 and 72 h. At the end of incubation, the drug-containing medium was replaced by 0.1 mL fresh medium, and 0.5 mg/ml MTT was added to each well, then incubated for 4 h. The formazan was dissolved in 150 μl dimethyl sulphoxide. The MTT absorbance was then read using a plate reader (Multiskan spectrum, Thermo Labsystems, OH, USA) at 570 nm. The cell viability was calculated as (OD^experiment^ – OD^blank^)/(OD^control^ – OD^blank^) × 100%.

### 2.4. Cell morphology

HK-2 cells were equally seeded into 6-well plates, and then exposed to mannitol at the concentrations of 0, 100, 250 mmol/L, respectively, for 48 h. After treatment for 48 h, the cells were observed under an inverted phase contrast microscope (Olympus, Tokyo, Japan) to investigate the morphological changes.

### 2.5. Apoptosis detection

The apoptosis rate of HK-2 cells was evaluated by Annexin V-FITC/PI double staining assay. The cells were treated with mannitol at the concentrations of 0, 100, 250 mmol/L for 48 h, respectively, then they were collected and resuspended in Annexin V-FITC/PI binding buffer for 10 min at room temperature in dark. Staining cells simultaneously with FITC Annexin V (green fluorescence) and the non-vital dye PI (propidium iodide; red fluorescence) allow the discrimination of intact cells (FITC^−^PI2^−^), early apoptotic (FITC^+^PI2^−^), late apoptotic (FITC^+^PI^+^) and necrotic cells (PI^+^). All experiments were repeated three times.

### 2.6. Measurement of GSH and MDA

HK-2 cells were cultured in 75 cm^2^ culture flask and exposed to various concentrations of mannitol (0, 100, 250 mmol/L) for different time duration of 24, 48 and 72 h, respectively. After exposure, cells were harvested and fractured by ultrasonication. The homogenate was then subjected to GSH and MDA determination by assay kits. The total protein concentration of cultured cells was determined by BCA assay kit. All procedures were performed according to the manufacturer’s instructions.

### 2.7. Fluorescence detection of F-actin

HK-2 cells were seeded at a density of 5 × 10^4^ cells/ml in 24-well plates chamber slides, then cells were treated with different concentrations of mannitol (0, 100, 250 mmol/L) for 24, 48, 72 h, respectively. After exposure, the cells were washed three times with phosphate-buffered saline, and fixed in a 4% paraformaldehyde for 30 min at 4 °C, then permeabilized with 0.2% Triton X-100 for 20 min and blocked with 0.5% BSA at room temperature for 10 min. Then, the cells were incubated with FITC-Phalloidin (5 g/ml) for 4 5 ∼ 60 min to localize F-actin filaments, and DAPI for 3–5 min in dark for nucleus staining. The cells were observed and photographed using a Carl Zeiss microscope.

### 2.8. Statistical analysis

All data were presented as mean ± SD (standard deviation). The comparison between groups was assessed by analysis of variance. *p* < .05 was considered statistically significant. Statistical analysis was performed with SPSS 19.0 software (IBM, Armonk, NY, USA).

## 3. Results

### 3.1. Mannitol inhibited the proliferation of HK-2 cells in a dose- and time-dependent manner

The anti-proliferation of mannitol on HK-2 cells was determined by MTT assay. The results indicated that mannitol inhibited the proliferation of HK-2 cells in a time- and dose-dependent manner (Table 1). Cell viability decreased significantly after treatment with 100–400 mmol/L mannitol for 24–72 h (*p* < .05). Based on these results and the related clinical reports, mannitol with concentrations of 100 mmol/L and 250 mmol/L were used in the following experiments.

**Table 1.** Effect of mannitol on HK-2 cell viability (%).

Groups	4 h	10 h	24 h	48 h	72 h
Con	100.0 ± 3.2	100.0 ± 3.3	100.0 ± 3.4	100.0 ± 1.2	100.0 ± 2.7
50 mM	98.9 ± 1.6	106.7 ± 1.3	94.7 ± 1.3	100.9 ± 3.7	87.2 ± 3.6*
100 mM	104.8 ± 2.4	102.9 ± 2.8	94.4 ± 2.5*	87.8 ± 1.5*	76.8 ± 2.1*
150 mM	100.4 ± 2.6	92.1 ± 3.3	90.6 ± 2.8*	74.9 ± 2.5*	67.5 ± 2.3*
200 mM	105.4 ± 1.2	92.2 ± 3.7*	82.7 ± 3.0*	62.5 ± 2.5*	61.1 ± 2.8*
250 mM	98.9 ± 3.1	96.2 ± 3.5*	79.5 ± 3.4*	58.3 ± 1.8*	57.2 ± 1.6*
300 mM	84.3 ± 2.4*	86.2 ± 3.0*	72.7 ± 4.0*	51.5 ± 3.5*	46.7 ± 1.7*
400 mM	84.0 ± 3.2*	66.1 ± 3.4*	66.0 ± 4.9*	42.1 ± 3.4*	28.7 ± 2.2*

(mean ± SD, *n* = 6).

* *p<*.05, compared with control group.

### 3.2. The changes of cell morphology

**Figure 1.**
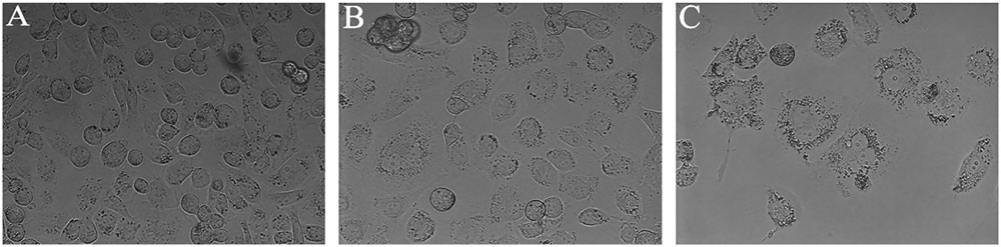
Morphological changes of HK-2 cells after treated with mannitol for 48 h. The cells were observed under an inverted phase contrast microscope with 400 magnification. (A) In control group, cells appeared in a spreading spindle shape. (B, C) After exposing HK-2 cells to 100 or 250 mM for 48 h, the cells became progressively swelling, membrane ruptured and detached. In addition, a remarkable decreased cell density was noticed.

HK-2 cells appeared in a spreading spindle shape in control group (Figure 1(A)). While, after treatment with 100 and 250 mmol/L mannitol for 48 h, the cells became progressively swelling, membrane ruptured and detached (Figure 1(B,C)).

### 3.3. Mannitol induced oxidative stress in HK-2 cells

**Figure 2.**
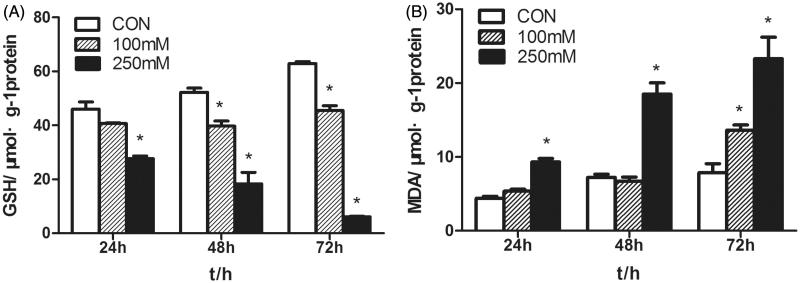
Effects of mannitol on GSH and MDA levels in HK-2 cells. (A) The content of GSH in the HK-2 cells treated with various concentrations of mannitol for 24, 48, 72 h. (B) The MDA levels of the HK-2 cells treated with various concentrations of mannitol for 24, 48, 72 h. Note: Compared with the control group, ‘*’ indicated significant difference (*p* < .05).

Lipid peroxidation was determined by measuring MDA concentration. Incubation of HK-2 cells with 250 mmol/L mannitol resulted in a dose- and time-dependent increase in MDA levels. While, the content of GSH decreased significantly, in 250 mmol/L mannitol group (18.3 ± 4.3 μmol/g) compared with that in control group (52.2 ± 1.6 μmol/g) (*p* < .05) (Figure 2).

### 3.4. Mannitol-induced apoptosis of HK-2 cells

**Figure 3.**
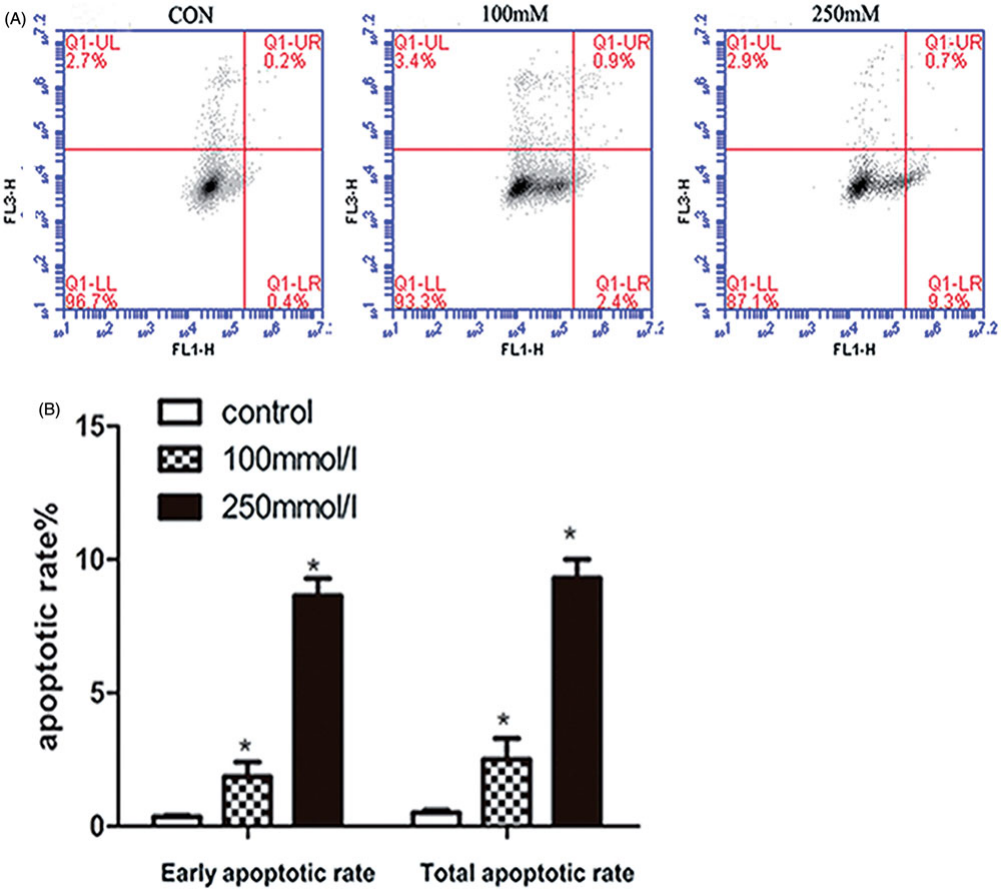
Effects of mannitol on the apoptosis of HK-2 cells. Flow cytometry assays showed apoptosis of HK-2 cells treated with 0, 100, 250 mM mannitol for 48 h. (A) Shown are representative dot plot of cells stained with Annexin V-FITC/PI following treatment with mannitol for 48 h. (B) Bars represent the cell percentages of early and total apoptotic cells after treatment with different concentrations of mannitol. Note: Compared with the control group, ‘*’ indicated significant difference (*p* < .05).

The results of flow cytometry showed that the total apoptosis rate was 0.5 ± 0.1% in control group, 2.5 ± 1.1% and 9.3 ± 1.0% in 100 and 250 mmol/L groups for 48 h, respectively. The increase of apoptosis rate was observed in HK-2 cells after treated with 250 mmol/L mannitol, compared with control group and 100 mmol/L group (*p* < .05) (Figure 3(B)).

### 3.5. Effects of mannitol on the cytoskeleton

**Figure 4.**
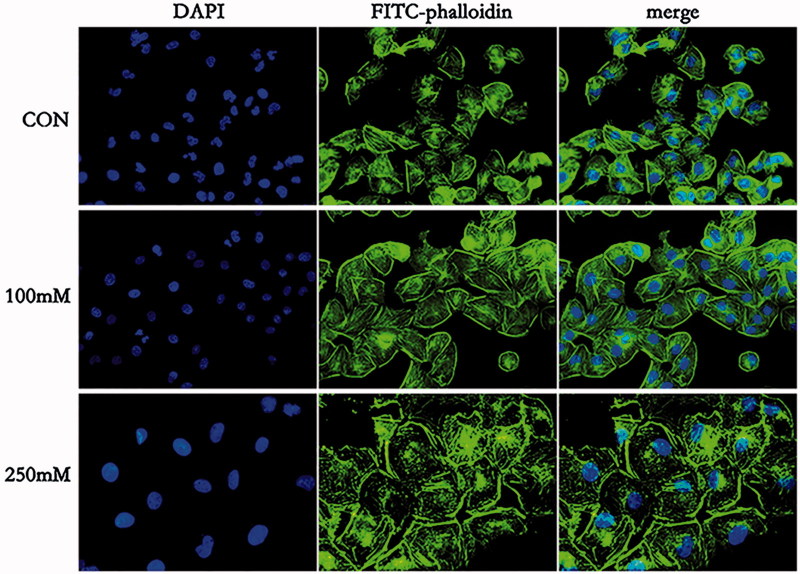
The cytoskeleton changes of HK-2 cells after treatment with mannitol for 48 h (×400). F-actin was visualized using FITC-phalloidin and cell nucleus was labeled by DAPI, respectively. Merged images of FITC-phalloidin and DAPI are also shown (merge).

To determine whether mannitol could induce F-actin rearrangement, immunofluorescence analysis was performed. In control group, the actin microfilament appeared well arranged and distributed in parallel lines along the axis of HK-2 cells. While after exposing the cells to 100 mmol/L mannitol for 48 h, actin microfilament became thinner, tense and disordered. Moreover, after exposure to 250 mmol/L mannitol for 48 h, F-actin in HK-2 cells showed disorganized and actin microfilament accumulated near the cell membrane (Figure 4).

## 4. Discussion

Mannitol-induced ARF has been reported and caused widely concerns in clinic. Although recent studies have reported that mannitol-induced nephrotoxicity *in vivo*, the exact mechanism remains unclear [19,20]. In the present work, we confirmed the cytotoxicity of mannitol on HK-2 cells *in vitro* by MTT and morphological observation. 100–250 mmol/L (amount to 18.22–45.54 g/L) mannitol significantly decreased viability of HK-2 cells in a time-dependent manner. This concentration was reported to be present in blood plasma after administration high dose of mannitol (2900 mg/dl) to dog [21].

The results showed that intracellular content of GSH decreased, while MDA level increased after mannitol treatment, suggesting that mannitol might exert its cytotoxicity via oxidative injury. Numerous studies revealed that oxidative stress marker was involved in the drug-induced nephrotoxicity [22]. GSH is the major intracellular antioxidant, the remarkable decrease in GSH induced by mannitol treatment suggested that the anti-oxidative potency was impaired. Reduced production of GSH would increase the sensitivity of cells to reactive oxygen species (ROS) and resulted in cellular oxidative injury [23]. GSH is capable of preventing damage to important cellular components caused by reactive oxygen species such as free radicals, peroxides, lipid peroxides and heavy metals. However, after the cellular GSH stores are depleted, oxygen free radicals and other toxic substances will accumulate to damage the biofilm system and intracellular oxidative phosphorylation [24]. Increased cell MDA contents in mannitol treatment groups demonstrated that the level of membrane lipid peroxidation elevated. MDA is the production of ROS in the biological cell membrane, MDA level increased remarkably suggesting the antioxidant-oxidant balance was destroyed. And this is consistent with the observed decrease of GSH content.

Accumulated evidence demonstrated that ROS played an important role in several models of apoptosis, the decrease in GSH content is a potential early activation signal for apoptosis, followed by ROS-induced cell apoptosis [25,26]. During the last few years, apoptosis was considered to be involved in drug-induced nephrotoxicity, which could result in renal damage [27]. Our results indicated that 100 and 250 mmol/L mannitol treatment could induce apoptosis, with apoptotic rate of 2.5 ± 1.1% and 9.3 ± 1.0%, respectively. These results suggested that apoptosis may contribute to mannitol-induced renal injury. However, the apoptosis rate was relatively low (9.3 ± 1.0%) in 250 mmol/L group for 48 h, while the cell viability decreased to 58.3 ± 1.8%, suggesting that other type of cell death independent of apoptosis might be involved in mannitol-induced renal injury. It was recently reported that ferroptosis was associated with GSH depletion, therefore, whether ferroptosis was involved in mannitol-induced cell injury is worthy of further investigation [28,29].

Our present work also showed that mannitol treatment caused cell morphological changes. Cytoskeleton is critical for cell movement, adhesion and structure foundation. Therefore, cytoskeleton was observed in our work by F-actin staining. We found that 100–250 mmol/L mannitol treatments resulted in cytoskeleton disorganization in HK-2 cells. These results demonstrated that mannitol treatment resulted in fiber breakage, accumulation of F-actin near the cell membrane and depolymerization eventually. In recent years, a large number of studies have shown that the disruption of cytoskeleton might directly affect pathological process of cell injury, and therefore contribute to renal disease [30]. For example, cisplatin [31] and endotoxin [32] affected the renal cytoskeleton protein expression and distribution, and eventually led to cell dysfunction and even cell death. Therefore, mannitol-induced cytoskeleton destruction might be involved in the development of ARF. Meanwhile, several studies have reported that oxidative stress produces a severe disruption of the microfilament cytoskeleton characterized by the fragmentation and patching of F-actin. However, the mechanisms of the cytoskeleton disruption by oxidative stress are unclear and may involve ATP depletion, oxidation of actin SH group and cross-linking of actin filaments [33–36].

Lots of studies have shown that intake of toxic agents may disturb antioxidant defense, the exact mechanism of antioxidant-oxidant imbalance induced by mannitol, needs further investigations.

In summary, the present work demonstrated that mannitol could induce renal tubular epithelial cells injury by increase in oxidative stress, inducing cell apoptosis and cytoskeleton destruction.
